# The changing returns to physical exercise in China (2010–2023): a nine-wave repeated cross-sectional study

**DOI:** 10.3389/fpsyg.2026.1912354

**Published:** 2026-10-05

**Authors:** Lamei Deng, Zhicheng Wang, Wei Liu, Chenyong Wei

**Affiliations:** 1 School of Humanities and Social Sciences, Xi’an Jiaotong University, Xi’an, China; 2 Hangzhou Medical College, Hangzhou, China; 3 Anhui Professional & Technical Institute of Athletics, Hefei, China

**Keywords:** China, institutional change, physical exercise, returns to exercise, Sports Power strategy, time trends

## Abstract

**Background:**

Since 2008, when China began to implement its strategy of building a sports power, the institutional context of physical exercise has changed substantially. Yet the temporal dynamics of the socioeconomic correlates of exercise remain underexplored.

**Objectives:**

To examine whether and how physical exercise participation and its associations with income, self-rated health, and social trust changed over the 2010–2023 study period.

**Methods:**

Using nine waves of the Chinese General Social Survey (CGSS 2010–2023, *N* = 78,467), we estimate annual marginal effects of exercise through ordinary least squares regression with exercise-by-year interactions. Three policy periods corresponding to phases of the Sports Power strategy are compared.

**Results:**

Exercise participation rose from 31.68% in 2010 to 51.26% in 2023. The exercise–income association increased from 0.301 (Period 1, 2010–2012) to 0.367 (Period 3, 2018–2023, +21.9%), although formal cross-period tests did not reach statistical significance; the upward trend is therefore only weakly consistent with Hypothesis 1. The exercise–health association was negative and appeared to deepen across periods, but the cross-period differences lost statistical significance once the non-comparable 2011 wave was excluded, so this apparent change is interpreted cautiously. The exercise–trust association moved from a non-significant −0.013 to −0.101 (SE = 0.014). Subgroup analyses showed larger income associations for men than women (0.420 versus 0.279) and for rural than urban residents (0.449 versus 0.180).

**Conclusion:**

Exercise participation rose markedly over the study period, and the exercise–trust association turned increasingly negative, but the exercise–income association did not strengthen to a statistically significant degree, and the health-selection pattern was sensitive to the exclusion of the 2011 wave. The observed changes are read as population-level associations rather than causal effects of the Sports Power strategy.

## Introduction

Physical activity is not merely a health-related behavior; it is also embedded in the structure of social hierarchy. The economic, physical, and social benefits of exercise depend on who exercises, where, and under what institutional conditions. China’s post-2008 Sports Power strategy provides an informative institutional setting for examining how institutional change relates to the socioeconomic correlates of exercise participation.

Institutional change has a certain directionality. In 2008, President Hu Jintao called for moving China from a major sports country to a sports power (Hu, 2008). A series of policies followed: in 2014, the State Council issued a directive on accelerating the sports industry and making national fitness a policy priority (State Council of China, 2014), and in 2016 the Healthy China 2030 outline added physical activity to the package of health measures oriented toward public welfare (Central Committee of the Communist Party of China and State Council of China, 2016). The policy sequence runs to 2050, by which time China aims to have become a sports power, with the 2019 Outline for Building a Sports Power as a key milestone (General Office of the State Council of China, 2019). These policies also reshaped the normative and cultural-cognitive pillars of the institutional environment for exercise (Scott, 2014).

Sociologists have always regarded exercise as a symbol of social class (Shilling, 2012) and a way to change one type of capital into another. Bourdieu (1984) found that bodily practices are stratified, but they also help perpetuate stratification. The time and skill required for physical exercise indicate economic and cultural capital, yet embodied capital acquired through regular exercise can in turn be converted into economic or social capital. In today’s China, an emerging economy, people with little money or education may be barred from many forms of physical activity, yet these activities could offer them opportunities to improve their status through effort.

Bourdieu’s theory of capital conversion allows us to examine exercise from a stratification perspective. Embodied capital, as defined by Bourdieu (1984), is cultural capital acquired through socialization and bodily practice. Our focus is not a single snapshot at one moment but how these dynamics evolve over time.

In China, Peng (2012) documented marked social gradients in exercise participation, finding that individuals with higher education and income participate more frequently. Fang and Guo (2019) showed that exercise may promote health equity, and Zhang et al. (2019) reported positive social-capital effects of exercise. Recent studies confirm that income and subjective class identity both shape who takes part in physical activity (Dong and Liu, 2022; Yang et al., 2022), and work on adolescents identifies family income as one barrier to participation (Durmusoglu et al., 2023). Globally, the World Health Organization [WHO] (2022) estimates that more than a quarter of adults are insufficiently active, with participation unevenly distributed across social groups.

This literature has two shared limitations. Nearly all of these studies draw on a single cross-sectional survey, so the associations between exercise and income, health, or social capital are treated as fixed quantities. They also pay little attention to the institutional environment, which means they cannot say whether these associations shift when national policy changes. What is missing is evidence on how exercise-related associations evolve over time under a changing policy regime. The present study addresses this gap by tracking the associations of exercise with income, self-rated health, and social trust across nine CGSS waves from 2010 to 2023, a window that covers the rollout and maturation of the Sports Power strategy. The study thereby links institutional change theory to health-related behavior in a non-Western setting, and its wave-by-wave estimates supply temporal evidence that single-wave designs cannot provide.

The present study shifts the analytic focus from a static participation-outcome correlation to a dynamic, institution-based framework. It addresses a central question in sports sociology—how institutions shape bodies and their social consequences—by measuring the associations of exercise with income, health, and social trust over time. China, as a rapidly institutionalizing economy, offers a well-suited setting for evaluating whether institutional change theory applies to health-related behavior.

We address three questions: (1) Did sports participation rates change from 2010 to 2023? (2) Did the associations between exercise and income, self-rated health, and social trust change over this period? (3) Are these changes consistent with distinct policy periods? Answering these questions sheds light on whether the Sports Power strategy coincided with broad shifts in the social patterning of exercise, moving beyond a single snapshot to a dynamic, over-time perspective.

These questions also bear on policy. The 2022 Beijing Winter Olympics brought sports back to the center of public policy and further promoted national fitness. The COVID-19 pandemic encouraged home-based and individualized exercise while restricting outdoor and collective activity (Stockwell et al., 2021). Studies conducted during the pandemic report only moderate happiness and life satisfaction among coaches and recreational exercisers (Atilgan, 2020; Atilgan and Tukel, 2021; Atilgan and Aksoy, 2021), with potential implications for social trust. A comprehensive review of participation levels and exercise-related associations from 2010 to 2023 can inform the evaluation of past sports policies and their adjustment after the pandemic.

## Theoretical framework and hypotheses

### Institutional change and the dynamic returns to exercise

North’s (1990) theory of institutional change is the foundation for our theoretical framework. Institutions change over time, and they alter the incentives and meanings attached to particular behaviors in a society. Scott (2014) identified three pillars of institutions—regulative, normative, and cultural-cognitive—each operating at multiple levels. In the case of physical exercise, the Sports Power strategy touched all three: regulation drove fitness infrastructure and policy directions (Hu and Fang, 2016); norms changed whether exercise is seen as a private activity or a socially encouraged practice; and cultural change made an active lifestyle more highly valued. Such institutional changes should ease the conversion of exercise into other forms of capital.

From the point of view of human capital (Becker, 1964; Grossman, 1972), exercise is a kind of investment in health. Regular exercise can improve fitness and lower the risk of disease, thereby improving labor productivity and earnings (Warburton et al., 2006; Rooth, 2011). The institutional environment will determine the rate of return from this investment. Where sports facilities are plentiful and fitness culture is strong, exercising translates into better health and then higher earnings more smoothly.

Institutional change can raise the economic returns from exercise by various means. One is signaling: a personal act becomes nationally encouraged, and participation turns into a positive social signal; it shows that you are disciplined, aware of your health, and lead an active lifestyle. Employers and colleagues may notice, influencing employment, promotions, and wages. Gyms, sports clubs, and running events are increasingly becoming places to socialize; exercisers build wider networks of their own and pick up all kinds of information and opportunities. Complementarity, the expansion of the sports industry, means exercisers may have more chances to find some sports-related work or part-time job that brings direct income.

Hypothesis 1 (H1): The income returns to physical exercise grew from 2010 to 2023.

### Institutional diffusion of exercise participation

Rogers’ (2003) diffusion of innovations theory is also relevant here. New practices spread through a society in an S-shaped pattern. Early adopters are usually well-educated and high-income innovators. As institutional support and infrastructure improve, adoption accelerates and the participant base widens. The Sports Power strategy is precisely this kind of institutional diffusion of exercise as a health innovation. At the individual level, participation depends on perceived benefits relative to costs. Public sports facilities and promoting a fitness culture reduce the time and money costs to participate while increasing the perceived benefits. At the system level, the speed of diffusion depends on institutional support and infrastructure.

Hypothesis 2 (H2): Exercise participation rates of Chinese urban and rural residents increased from 2010 to 2023.

### Health selection pattern and social trust

The relationship between exercise and self-rated health is subject to a selection bias: people who perceive themselves as having poor health might start exercising or do more, resulting in a negative statistical link in cross-sectional data (Lechner, 2009). For social trust, Putnam (2000) argued that the effect of exercise on social capital depends on how organized the activity is. Collective sports lead to interaction and trust, but solo fitness—running alone, using a fitness app—could replace face-to-face social contact.

Hypothesis 3a (H3a): The relationship between exercise and self-rated health is negative, and this negative association may intensify across periods if the participant pool shifts toward health-concerned individuals.

Hypothesis 3b (H3b): There is a time dimension to the relationship between exercise and social trust: as individual fitness practices become more common, the negative relationship gets stronger.

### Heterogeneity in returns: gender and urban-rural divides

Returns to exercise may differ systematically by social position. Gender role expectations in China allocate disproportionate domestic responsibilities to women, creating time poverty that could constrain both participation quality and the realization of returns. Urban-rural differences in sports infrastructure and fitness culture may also shape returns. We treat gender and urban-rural status as exploratory dimensions. Women’s exercise returns may be squeezed by the dual burden of time scarcity and labor market discrimination, while rural residents’ returns may benefit from the scarcity premium of exercise as a positive signal where participation is less common.

## Materials and methods

### Data source

We use nine waves of the Chinese General Social Survey (CGSS) from 2010 through 2023 (2010, 2011, 2012, 2013, 2015, 2017, 2018, 2021, and 2023). The CGSS is administered by the Survey Research Center of Renmin University of China using a multi-stage stratified probability sampling design covering all mainland provinces. After dropping the 2008 wave (which lacked the exercise variable) and removing observations with missing values on key covariates, the pooled dataset contains 82,480 observations. Of these, 78,470 have valid exercise information (the participation sample), and 78,467 enter the income and health regressions after additionally requiring non-missing urban status; the social-trust regressions use 78,280 observations. Wave sizes range from 5,090 (2011) to 10,786 (2012). Each wave interviews a fresh sample of respondents, so the data are repeated cross-sections rather than a panel: the same individuals are not followed over time, and individual-level change cannot be measured. The temporal comparisons reported below therefore describe population-level associations, not within-person change.

### Variable definitions

Three dependent variables capture the multidimensional correlates of exercise. The economic outcome is measured as the natural logarithm of annual personal income, lnincome = ln(income + 1), which accommodates zero income and corrects right skew. In the original CGSS, self-rated health is coded on a five-point scale from 1 (very healthy) to 5 (very unhealthy); we reverse-coded it as health = 6−original value so that larger values indicate better health (range 1–5). Social trust is measured by agreement with the statement that most people can be trusted, originally coded from 1 (strongly agree) to 5 (strongly disagree); we reverse-coded it as trust_rev = 6−original value so that higher values indicate greater trust (range 1–5). Because the source item varies across waves (health: a15 in most waves, d26 in 2011, A15 in 2021; trust: a33 in 2010–2015 and 2023, v458 in 2017, v505 in 2018, A33 in 2021), we applied the same reverse-coding rule to every wave to ensure comparability.

The key independent variable is physical exercise participation (exercise). The CGSS asks respondents about their frequency of physical exercise in the past year, with five response categories: (1) never, (2) rarely, (3) sometimes, (4) often, and (5) very frequently. Respondents answering sometimes, often, or very frequently were classified as exercisers (exercise = 1); those answering never or rarely were classified as non-exercisers (exercise = 0). The same rule held for every survey wave.

Control variables include age and its square (age, age^2^), to capture non-linear life-cycle effects; gender (female = 1); years of education (educ), converted from categorical educational attainment to continuous years; urban hukou registration (urban = 1); and year fixed effects to control for macroeconomic and societal variations across survey years.

### Analytical strategy

We conduct a policy-period analysis by dividing the observation window into three periods aligned with the evolution of the Sports Power strategy: Period 1 (2010–2012), the concept-initiation phase when the strategy was first announced; Period 2 (2013–2017), the institution-building phase when national fitness was elevated to a national strategy and infrastructure expanded rapidly; and Period 3 (2018–2023), the institutional-maturation phase. Period 3 begins in 2018 because the 19th National Congress of the CPC (2017) called for accelerating the building of a sports power, and the sports-power agenda was formalized in the 2019 Outline for Building a Sports Power; we additionally test the sensitivity of the results to an alternative 2019 cut-point. Each period regression includes year fixed effects, and standard errors are Huber–White heteroskedasticity-consistent estimates. To assess whether the exercise–outcome associations differ across periods, we estimate unified exercise-by-period interaction models and report formal Wald (contrast) tests. Sampling weights are available for each wave but differ in construction across waves, so the main analyses are unweighted; a weighted sensitivity analysis is reported alongside the primary results.

## Results

### Trends in exercise participation

Table 1 reports the yearly exercise participation rate in the CGSS sample. Participation rose from 31.68% in 2010 to 51.26% in 2023, an increase of 19.58 percentage points. Participation held relatively steady at around 31%–33% during 2010–2013. Participation then rose by 12.45 percentage points between the 2013 and 2015 waves (from 30.90% to 43.35%), coinciding with the deployment of national fitness policy. Growth continued after 2017 at a slower pace, reaching roughly 51% by 2023. The 2023 rate (51.26%) was slightly below the 2021 rate (55.30%), which may reflect post-pandemic changes in exercise behavior.

**TABLE 1:** Physical exercise participation rates by year, Chinese General Social Survey (CGSS) 2010–2023.

Survey year	*N*	Non-exercise (%)	Exercise (%)	Cumulative change (pp)
2010	10,509	68.32	31.68	–
2011	5,090	67.07	32.93	+1.25
2012	10,786	69.10	30.90	−0.78
2013	10,227	69.10	30.90	−0.78
2015	8,673	56.65	43.35	+11.67
2017	10,553	55.61	44.39	+12.71
2018	10,704	52.31	47.69	+16.01
2021	6,033	44.70	55.30	+23.62
2023	5,895	48.74	51.26	+19.58

Exercise participation is defined as engaging in exercise at least sometimes in the past year. Cumulative change is calculated relative to the 2010 baseline (percentage points).

The overall rise masked a clear urban–rural divide. Participation was consistently higher in urban than in rural areas, and the two groups followed different trajectories: the urban rate was already 48.5% in 2010 and reached 70.5% in 2021 before easing to 65.6% in 2023, whereas the rural rate started at 18.2% in 2010 and climbed more steeply after 2015, reaching 51.2% in 2021 and 47.2% in 2023. A formal test of the year-by-urban/rural interaction was highly significant (chi-square = 299.68, df = 8, *p* < 0.001), confirming that the urban and rural participation paths diverged over the study period.

This pattern is broadly consistent with Rogers’ (2003) diffusion-of-innovations theory. The 2010–2013 plateau resembles the slow-diffusion phase among early adopters, and the 2015 increase coincides with intensified national-fitness policy deployment following the 2014 State Council directive. The slower growth after 2017 is descriptively consistent with a diffusion curve approaching saturation, although no formal breakpoint test was performed.

### Exercise–income associations

Table 2 reports the average marginal effects of exercise on log income in the exercise-by-year interaction model. Exercise participation was positively associated with income in all years (all *p* < 0.001), but the strength of this association showed large variation across different years and different phases.

**TABLE 2:** Average marginal effects of exercise on log income by year.

Year	Marginal effect	Robust SE	*t*	*P*	95% CI
2010	0.441	0.053	8.37	<0.001	[0.338, 0.544]
2011	0.387	0.068	5.68	<0.001	[0.253, 0.520]
2012	0.175	0.044	3.97	<0.001	[0.089, 0.260]
2013	0.398	0.047	8.49	<0.001	[0.306, 0.490]
2015	0.291	0.024	12.24	<0.001	[0.244, 0.337]
2017	0.376	0.036	10.48	<0.001	[0.305, 0.447]
2018	0.425	0.035	12.04	<0.001	[0.356, 0.494]
2021	0.259	0.059	4.43	<0.001	[0.144, 0.374]
2023	0.359	0.046	7.82	<0.001	[0.269, 0.449]

From the exercise-by-year interaction OLS model controlling for age, age squared, gender, education, urban hukou, and year fixed effects.

The marginal effect in 2010 is 0.441 (SE = 0.053), the highest across the whole observation period. This strong association also partly reflects an elite selection pattern: In 2010, most of those participating in physical activity came from groups with high levels of education and income. The year 2012 experienced a noticeable low point where the marginal effect plummeted to 0.175 (SE = 0.044), the lowest level recorded throughout the period. The slowdown in China’s GDP growth rate at that time was accompanied by a decline in participation rates for physical activity. The income association fluctuated between 2013 and 2018. By 2013, the marginal effect had risen again to 0.398 (SE = 0.047). It fell to 0.291 (SE = 0.024) in 2015, climbed to 0.376 (SE = 0.036) in 2017, and rose further to 0.425 (SE = 0.035) in 2018.

The 2018–2023’s V-shaped path stands out. The association was 0.425 in 2018, dropped to 0.259 (SE = 0.059) in 2021, then rose to 0.359 (SE = 0.046) in 2023. The decline in 2021 may reflect labor-market disruptions during the COVID-19 outbreak; the recovery in 2023 may indicate that the exercise–income association rebounded as labor-market conditions normalized after the pandemic.

### Exercise–health associations and the 2011 anomaly

Table 3 reports the yearly marginal effects of exercise on self-rated health. Except for 2011, the coefficients are negative and statistically significant (range: −0.126 to −0.277; all *p* < 0.001). Exercisers report lower self-rated health than non-exercisers after controlling for sociodemographic covariates. This counterintuitive result reflects a health selection pattern: people in poorer health are more likely to start or increase exercise.

**TABLE 3:** Average marginal effects of exercise on self-rated health by year.

Year	Marginal effect	Robust SE	*t*	*P*	95% CI
2010	−0.165	0.020	−8.03	<0.001	[−0.205, −0.124]
2011	+0.320	0.038	8.35	<0.001	[0.245, 0.395]
2012	−0.179	0.019	−9.17	<0.001	[−0.217, −0.140]
2013	−0.126	0.020	−6.27	<0.001	[−0.165, −0.086]
2015	−0.155	0.021	−7.42	<0.001	[−0.196, −0.114]
2017	−0.277	0.019	−14.60	<0.001	[−0.315, −0.240]
2018	−0.207	0.019	−10.96	<0.001	[−0.244, −0.170]
2021	−0.186	0.026	−7.09	<0.001	[−0.237, −0.134]
2023	−0.205	0.027	−7.59	<0.001	[−0.257, −0.152]

The 2011 is an anomalous positive value, likely attributable to differences in health measurement items across survey waves.

The 2011 anomaly most likely stems from differences in survey instrumentation: CGSS 2011 used a different health-module item (d26) than all other waves (a15), so subtle wording and anchoring differences may have introduced systematic measurement shifts. In a sensitivity analysis excluding 2011 using the same period-specific specification as Table 5, the Period 1 health coefficient changed from −0.071 to −0.130 (SE = 0.015), converging toward the Period 2 (−0.162) and Period 3 (−0.214) estimates (Table 7B). In the unified interaction model the cross-period differences were no longer statistically significant (Period 2 vs. Period 1: −0.018, *p* = 0.319; Period 3 vs. Period 1: −0.024, *p* = 0.220; omnibus exercise-by-period test, F = 0.82, *p* = 0.440), indicating that the apparent strengthening of the health selection pattern is largely driven by the 2011 anomaly. We therefore interpret the 2011 estimate primarily as a measurement-comparability issue rather than sampling instability.

In the main period-specific estimates (Table 5), the health selection coefficient moved from −0.071 (SE = 0.015) in Period 1 to −0.162 (SE = 0.012) in Period 2 and −0.214 (SE = 0.014) in Period 3. This apparent strengthening, however, is not robust to the exclusion of the anomalous 2011 wave: the cross-period differences lose statistical significance once 2011 is removed, so the intensification of the health selection pattern should be interpreted cautiously.

### Exercise–trust associations

Table 4 reports the annual marginal effects of exercise on social trust. From 2010 to 2013, there is no significant association between exercising and trusting (2010: +0.036, *p* = 0.115; 2011: −0.034, *p* = 0.275; 2012: −0.022, *p* = 0.291; 2013: −0.041, *p* = 0.068). In 2015, a sizable negative relationship began and continued to grow stronger over time (2015: −0.076, *p* < 0.001; 2018: −0.092, *p* < 0.001; 2021: −0.121, *p* < 0.001; 2023: −0.138, *p* < 0.001). In 2017 the association rebounded slightly (−0.025, *p* = 0.219), but from 2015 onward the negative association deepened over time.

**TABLE 4:** Average marginal effects of exercise on social trust by year.

Year	Marginal effect	Robust SE	*t*	*P*	95% CI
2010	0.036	0.023	1.57	0.115	[−0.009, 0.082]
2011	−0.034	0.032	−1.09	0.275	[−0.096, 0.027]
2012	−0.022	0.021	−1.06	0.291	[−0.064, 0.019]
2013	−0.041	0.022	−1.83	0.068	[−0.084, 0.003]
2015	−0.076	0.021	−3.71	<0.001	[−0.116, −0.036]
2017	−0.025	0.020	−1.23	0.219	[−0.064, 0.015]
2018	−0.092	0.019	−4.76	<0.001	[−0.130, −0.054]
2021	−0.121	0.026	−4.74	<0.001	[−0.172, −0.071]
2023	−0.138	0.027	−5.15	<0.001	[−0.191, −0.086]

Negative association became substantial from 2015 onward, coinciding with the rise of individualized fitness practices.

**TABLE 5:** Exercise associations with income, self-rated health, and social trust by policy period.

Outcome	Period 1 (2010–2012)	Period 2 (2013–2017)	Period 3 (2018–2023)	Direction
Log income	0.301*** (0.033)	0.314*** (0.022)	0.367*** (0.026)	Rising
Self-rated health	−0.071*** (0.015)	−0.162*** (0.012)	−0.214*** (0.014)	Worsening
Social trust	−0.013 (0.015)	−0.043*** (0.013)	−0.101*** (0.014)	Worsening

Coefficients from period-specific OLS regressions controlling for age, age squared, gender, education, urban hukou, and year fixed effects. Robust standard errors in parentheses. ***P < 0.01.

**TABLE 6:** Pooled exercise–income associations by gender and urban-rural status.

Group	Coefficient	SE	*N*	R^2^
Male	0.420***	0.020	40,184	0.143
Female	0.279***	0.024	38,283	0.109
Urban	0.180***	0.023	27,104	0.087
Rural	0.449***	0.021	51,363	0.112

Each regression controls for age, age-squared, education, and year fixed effects. Gender models additionally control for urban hukou; urban-rural models additionally control for gender. ***P < 0.01.

**TABLE 7:** Robustness checks.

A. Robustness of the exercise–income association to alternative income specifications				
**Specification**	**Period 1**	**Period 2 vs. 1**	**Period 3 vs. 1**	***N***
Main (ln income)	0.322*** (0.031)	0.034 (0.037)	0.042 (0.040)	78,467
Positive income	0.322*** (0.031)	0.034 (0.037)	0.042 (0.040)	78,467
Winsorized (1%/99%)	0.320*** (0.031)	0.034 (0.037)	0.043 (0.040)	78,467
Trim top 1%	0.322*** (0.031)	0.034 (0.037)	0.042 (0.040)	78,467
**B. Self-rated health association with exercise, with and without the 2011 wave**				
**Specification**		**Period 1 (2010–2012)**	**Period 2 (2013–2017)**	**Period 3 (2018–2023)**
Including 2011		−0.071*** (0.015)	−0.162*** (0.012)	−0.214*** (0.014)
Excluding 2011		−0.130*** (0.015)	−0.162*** (0.012)	−0.214*** (0.014)
**C. Unified exercise-by-period interaction models for income, health, and trust**				
**Outcome**	**Period 1**	**Period 2 vs. 1**	**Period 3 vs. 1**	**Omnibus test**
Log income	0.322*** (0.031)	0.034 (0.037)	0.042 (0.040)	*F* = 0.60, *p* = 0.550
Self-rated health	−0.076*** (0.014)	−0.117*** (0.018)	−0.125*** (0.019)	*F* = 27.97, *p* < 0.001
Social trust	−0.001 (0.014)	−0.044** (0.018)	−0.111*** (0.019)	*F* = 16.76, *p* < 0.001

Coefficients from exercise-by-period interaction or period-specific OLS models controlling for age, age squared, gender, education, urban hukou, and year fixed effects. Robust standard errors in parentheses. In Panel A, the positive-income subsample is identical to the main specification because personal income is already positive for all respondents; in Panel B, after excluding 2011 the cross-period contrasts are no longer significant (Period 2 vs. Period 1: −0.018, *p* = 0.319; Period 3 vs. Period 1: −0.024, *p* = 0.220; omnibus F = 0.82, *p* = 0.440). In Panel C, the omnibus test is a joint Wald test of the two exercise-by-period interaction terms (df = 2). ***p < 0.01, **p < 0.05.

This temporal pattern is consistent with the rise of individualized fitness in China, although the CGSS does not record exercise type or whether exercise is performed individually or collectively. Around 2010, collective forms of exercise such as group dancing, workplace sports, and amateur team sports were common in Chinese communities. After 2015, smartphones and fitness apps may have made solo routines such as running alone, gym sessions, and home workouts more accessible. These contextual shifts may help explain why the strongest negative trust association appears in 2023 (−0.138, SE = 0.027), but they remain interpretations rather than measured mechanisms. Policy-period regressions show the same pattern: the trust coefficient moved from −0.013 (ns) in Period 1 to −0.043 (*p* < 0.01) in Period 2 and −0.101 (*p* < 0.001) in Period 3 (Table 5); an omnibus test of the exercise-by-period interaction was highly significant (F = 16.76, *p* < 0.001), confirming that the trust penalty attached to exercise grew over the study period.

### Policy period comparisons

Table 5 reports the regression results for each policy period. The exercise–income association rose from 0.301 (SE = 0.033) in Period 1 to 0.314 (SE = 0.022) in Period 2 and 0.367 (SE = 0.026) in Period 3, a cumulative increase of 21.9%. In a unified exercise-by-period interaction model, the period-specific associations were 0.322 (95% CI = 0.261, 0.383) in Period 1, 0.356 (95% CI = 0.313, 0.398) in Period 2, and 0.363 (95% CI = 0.312, 0.414) in Period 3. The Period 2 versus Period 1 contrast was 0.034 (*p* = 0.358) and the Period 3 versus Period 1 contrast was 0.042 (*p* = 0.299); an omnibus test of the exercise-by-period interaction did not reject the null (F = 0.60, *p* = 0.550). The upward trend is therefore only weakly consistent with Hypothesis 1. This pattern remains compatible with institutional change theory, which predicts that the economic value attached to a practice rises as the surrounding institutional environment strengthens. A sensitivity check using a 2019 cut-point (Period 2 = 2013–2018, Period 3 = 2021–2023) yielded a Period 3 coefficient of 0.309, slightly below the Period 1 estimate of 0.322 (difference = −0.014, *p* = 0.776), indicating that the upward trend in the exercise–income association is sensitive to the choice of cut-point.

In the period-specific estimates, the health coefficient moved from −0.071 (SE = 0.015) to −0.162 (SE = 0.012) and −0.214 (SE = 0.014). This apparent intensification is not robust, however: once the 2011 wave is removed, the period contrasts lose significance (see below), so the negative selection pattern should be treated as stable rather than clearly deepening. The exercise–trust association shifted from non-significance (−0.013, SE = 0.015) to a moderate negative coefficient (−0.043, SE = 0.013, *p* < 0.01) and then to a sizeable negative association (−0.101, SE = 0.014, *p* < 0.001). The trust penalty attached to exercise thus grew steadily across the three policy periods. Formal period-specific estimates, pairwise contrasts, and omnibus tests for all three outcomes are summarized in Table 7C.

### Subgroup analyses: gender and urban-rural differences

Table 6 reports pooled regression results by gender and urban-rural status. The exercise–income association for males was 0.420 (SE = 0.020, *p* < 0.01), considerably higher than for females (0.279, SE = 0.024, *p* < 0.01), a difference of 50.5%. This gender gap likely reflects a combination of women’s heavier domestic workload leaving them less time for exercise, and labor market discrimination depressing the earnings they realize from equal improvements in health capital.

The urban-rural comparison shows a particularly striking pattern: the exercise–income association for rural residents (0.449, SE = 0.021, *p* < 0.01) was approximately 2.5 times that for urban residents (0.180, SE = 0.023, *p* < 0.01). This rural premium operates through a diminishing marginal returns framework: where exercise is more prevalent (urban areas), its signaling value and capital conversion efficiency are lower; where it is less common (rural areas), the relative advantage of exercisers over non-exercisers is more pronounced. Rural labor markets in China tend to be more physically demanding, making the health capital generated through exercise more directly relevant to labor productivity and earnings.

In a unified interaction model, the exercise-by-gender interaction (−0.158, *p* < 0.001) and the exercise-by-urban/rural interaction (−0.269, *p* < 0.001) were both statistically significant, confirming that the observed gender and urban-rural differences reflect genuine heterogeneity rather than sampling variation.

Because the analyses are unweighted, we re-estimated the principal models with wave-specific sampling weights. Weighting left the exercise–income association in Period 1 somewhat larger (0.361 versus 0.322) and, more importantly, reversed the sign of the Period 3 interaction (from +0.042 to −0.029), further undercutting any claim of a rising income return. The health and trust results were largely unaffected by weighting, and weighted participation estimates were close to the unweighted ones.

To assess whether the income results are sensitive to the treatment of non-employed respondents and extreme values, we re-estimated the unified exercise-by-period income model under three alternative specifications. In the analytical sample, personal income is already positive for every respondent, so the +1 term in ln(income + 1) serves only to stabilize the log transformation and does not retain a zero-income subgroup; consequently, the positive-income subsample yields results identical to the main specification. We additionally winsorized income at the 1st and 99th percentiles before log transformation and, separately, dropped observations above the 99th percentile. Across all four specifications the Period 1 exercise coefficient was nearly unchanged (0.322, 0.322, 0.320, and 0.322), as were the interaction contrasts (Period 3 vs. Period 1: 0.042, 0.042, 0.043, and 0.042). The non-significant income trend is therefore not an artifact of zero-income inclusion or of a small number of extreme observations (Table 7A).

## Discussion

### Institutional change and the rising economic value of exercise

The period-specific estimates show an exercise–income association that rose from 0.301 in Period 1 to 0.367 in Period 3, but this increase did not reach statistical significance in the formal cross-period tests. This pattern is consistent with North’s (1990) view that institutional arrangements shape the economic importance of specific behaviors. In China, the strengthening association during the Sports Power era may reflect three complementary pathways: official endorsement may have raised the signaling value of exercise, infrastructure investment may have lowered entry barriers, and cultural promotion may have reinforced the perception of exercise as human-capital investment. However, these mechanisms are not directly measured.

This finding can also be compared with international evidence. Using German panel data, Lechner (2009) found that the labor-market association of sport participation remained fairly stable. The strengthening exercise–income association in China may imply that institutional change plays a larger role in shaping these associations in transitioning economies than in mature ones, where institutions are more stable. This is consistent with theoretical expectations that the rate of return on human capital depends on the institutional environment (Becker, 1964).

### The persistence of the health selection pattern

Instead of shrinking as participation expanded, the health selection pattern persisted across the three periods (from −0.071 to −0.214 in the period-specific estimates), although this apparent deepening is not robust to the exclusion of the 2011 wave. The shift in who participates probably drives the pattern. Early participants (2010–2012) were mostly high-education, high-income individuals who exercised for health maintenance—preventive motivation. New participants after 2015, particularly the large influx of middle-aged and lower-income groups, took up exercise largely because they already sensed their health was declining. This shift from preventive to reactive participation is one explanation for why the negative association deepened over time.

To provide observable evidence on the compositional mechanism, we compared the characteristics of exercisers across waves. The average age of exercisers rose from 47.5 years in 2010 to about 50.1 years by 2023, while their mean education fluctuated around 10–11 years without a clear upward trend; mean log income hovered near 10.6–10.9 across waves. The share of exercisers who were women remained roughly stable (44%–50%), but the urban-hukou share fell sharply from 68% in 2010 to about 28% by 2023, mirroring the steeper participation growth in rural areas. This shift in the composition of exercisers—toward older, increasingly rural participants—is consistent with the diffusion of exercise beyond its early, urban, advantaged adopters, though it should be read as descriptive rather than as a formal test of any single mechanism.

In policy terms, this means that raising participation rates alone is not enough to deliver the health benefits of exercise. Interventions need to address exercise quality—type, intensity, frequency, and consistency—not just participation rates. Targeted exercise prescriptions and health management services for health-concerned new participants could maximize the actual health benefits of exercise.

### Trust deficit and the individualization of fitness

The exercise–trust association moved from non-significant in the early years to a sizeable negative association in Period 3 (−0.101). This pattern may reflect the accelerating individualization of fitness in China—the shift from collective sports to phone-mediated, solo routines—although the data do not directly measure whether exercise is individual or collective. Putnam’s (2000) portrait of declining American social capital—the move from league bowling to bowling alone—offers a suggestive parallel to China’s contemporary fitness culture.

The weak rebound in 2017 (−0.025, ns) provides a suggestive contrast. Around that year, mass-participation events, city marathons, and orienteering gained temporary ground, which may have briefly restored the social dimension of exercise. But the deepening trust deficit after 2018 suggests the structural push toward individualized fitness has overwhelmed these episodic collective movements.

### The rural premium: exercise as a scarce social signal

Rural residents showed a larger exercise–income association (0.449) than urban residents (0.180), one of the study’s more surprising results. Several interpretations are plausible. First, scarcity signaling: where exercise is less common—as in rural China—participation may constitute a stronger signal of discipline, health, and modernity. Second, labor-market structure: rural labor tends to be more physically demanding, so health capital from exercise may translate more directly into productivity. Third, diminishing marginal returns: as exercise approaches universality in urban areas, its power to differentiate individuals—and thus its economic value—may decline. These mechanisms warrant direct testing in future research.

Implications for spatial equity of sports policy. Rural sports infrastructure investments might provide greater economic benefits per unit of investment because the starting point is lower, and exercise may carry greater signaling value and matter more for labor productivity in rural areas.

### Limitations and future directions

Several limitations should be considered. First, and most importantly, the CGSS is repeated cross-sectional data, so we cannot follow individuals over time or rule out unobserved heterogeneity, reverse causality, and omitted-variable bias. All coefficients reported here should therefore be interpreted as adjusted associations rather than causal effects. Panel datasets such as the China Family Panel Studies (CFPS) would permit individual fixed-effects estimation that better controls for time-constant confounders. Second, our main analyses are unweighted because the sampling weights differ in construction across waves; we report a weighted sensitivity analysis, and national-level estimates of participation and their uncertainty should still be read with some caution. Third, our dichotomous exercise measure ignores exercise type, intensity, frequency, and whether it is performed individually or collectively. Fourth, income is not adjusted for inflation across waves, which may affect the comparability of the income coefficients over time. Finally, the 2011 health anomaly underscores the importance of verifying measurement equivalence across waves in longitudinal comparisons.

Future work can proceed in different ways. Panel analysis following people’s own exercise trajectories would provide more credible causal estimates. Adding measures of the type and intensity of exercise may uncover how these associations vary by mode. Qualitative methods could capture the subjective experience, motivation, and perceived gains of various population groups, and thereby help explain the quantitative patterns. The further agenda relates to How digital fitness technology interacts with social capital; do online fitness communities replace or supplement the social bonding of traditional collective sports?

## Conclusion

Using nine waves of CGSS data from 2010 to 2023, we examined how exercise participation and its associations with income, self-rated health, and social trust changed over this period of institutional change in China. The main findings are as follows:

First, exercise participation increased from 31.68% to 51.26%, with a marked rise between the 2013 and 2015 waves that is descriptively consistent with Rogers’ (2003) institutional diffusion theory. Second, the exercise–income association fluctuated across years and showed no statistically significant rise across policy periods, so the data do not support a strengthening of the economic return to exercise over the Sports Power era. Third, the exercise–trust association turned increasingly negative over time, whereas the health selection pattern did not clearly intensify once the 2011 wave was excluded; together these patterns point to changes in the composition and organization of exercise participation in China. Fourth, the exercise–income association exhibits pronounced heterogeneity, being larger for men than women and for rural than urban residents.

These results carry policy implications. Sports investment may merit being accounted for as human capital; sports infrastructure is more than welfare spending and may function as physical capital with economic value. Policy evaluation should go beyond the single measure of participation rates to incorporate long-term, multi-dimensional outcomes. If future research confirms that individualized fitness erodes social trust, post-pandemic sports policy may consider supporting collective forms of exercise. Finally, the gender gap and the rural–urban difference suggest that equity should be considered so that the benefits of the Sports Power approach reach diverse population groups.

This study provides evidence on how institutional change is associated with the socioeconomic correlates of physical activity in a rapidly changing society: the Sports Power era coincided with rising participation, though not with a statistically significant strengthening of the exercise–income association. Yet the divergent trends in health selection and social trust imply that policy has many facets. Whether future sports governance in China succeeds hinges not only on raising participation but also on the type, quality, and distribution of the outcomes associated with people’s engagement in sport.

## Data Availability

The raw data supporting the conclusions of this article will be made available by the authors, without undue reservation.
